# First-In-Class Small Molecule ONC201 Induces DR5 and Cell Death in Tumor but Not Normal Cells to Provide a Wide Therapeutic Index as an Anti-Cancer Agent

**DOI:** 10.1371/journal.pone.0143082

**Published:** 2015-11-18

**Authors:** Joshua E. Allen, Roslyn Crowder, Wafik S. El-Deiry

**Affiliations:** 1 Oncoceutics, Inc., Philadelphia, Pennsylvania, 19103, United States of America; 2 Department of Biology, Stetson University, DeLand, Florida, 32723, United States of America; 3 Laboratory of Translational Oncology and Experimental Cancer Therapeutics, Department of Oncology and Molecular Therapeutics Program, Fox Chase Cancer Center, Philadelphia, Pennsylvania, 19111, United States of America; Emory University, UNITED STATES

## Abstract

We previously identified ONC201 (TIC10) as a first-in-class orally active small molecule with robust antitumor activity that is currently in clinical trials in advanced cancers. Here, we further investigate the safety characteristics of ONC201 in preclinical models that reveal an excellent safety profile at doses that exceed efficacious doses by 10-fold. In vitro studies indicated a strikingly different dose-response relationship when comparing tumor and normal cells where maximal effects are much stronger in tumor cells than in normal cells. In further support of a wide therapeutic index, investigation of tumor and normal cell responses under identical conditions demonstrated large apoptotic effects in tumor cells and modest anti-proliferative effects in normal cells that were non-apoptotic and reversible. Probing the underlying mechanism of apoptosis indicated that ONC201 does not induce DR5 in normal cells under conditions that induce DR5 in tumor cells; DR5 is a pro-apoptotic TRAIL receptor previously linked to the anti-tumor mechanism of ONC201. GLP toxicology studies in Sprague-Dawley rats and beagle dogs at therapeutic and exaggerated doses revealed no dose-limiting toxicities. Observations in both species at the highest doses were mild and reversible at doses above 10-fold the expected therapeutic dose. The no observed adverse event level (NOAEL) was ≥42 mg/kg in dogs and ≥125 mg/kg in rats, which both correspond to a human dose of approximately 1.25 g assuming standard allometric scaling. These results provided the rationale for the 125 mg starting dose in dose escalation clinical trials that began in 2015 in patients with advanced cancer.

## Introduction

ONC201, also known as TIC10, is a small molecule that is currently in phase II clinical trials in select advanced cancer indications. In preclinical studies, ONC201 possesses a cancer mutation-agnostic efficacy profile that is not impaired by common oncogenic mutations that drive disease progression and therapeutic resistance. We previously reported the identification of ONC201 as a novel first-in-class orally active small molecule with anti-cancer activity based on its ability to induce the pro-apoptotic TRAIL pathway [1]. The TRAIL pathway is a critical effector mechanism in the immune surveillance of cancer that is capable of eliminating tumor cells via apoptosis without harming normal host cells [2]. Immune cells upregulate TRAIL on their cell surface, which binds to the pro-apoptotic receptors, DR4 and DR5, that are expressed on the surface of tumor cells to ultimately trigger the extrinsic or intrinsic cell death pathway in a cell-type dependent manner [3].

Despite their original motivation, many targeted therapies have turned out to be significantly toxic and chemotherapies remain the cornerstone of medical intervention in oncology for most indications. Chemotherapeutic agents or targeted agents cause a host of severe side-effects that are debilitating and dose limiting, such as nephrotoxicity, hepatotoxicity, peripheral neuropathy, fatigue, nausea, anemia, skin toxicities, GI toxicities, lung toxicities, cardiac toxicities, leukoencephalopathy, thrombocytopenia and neutropenia among others [4]. Thus, there is an unmet need for treatments with fewer side-effects in oncology [5], which is especially pronounced for the cancer patient population who are disproportionally elderly, frail, suffer from irreversible therapy-related toxicities and/or would benefit from chronic therapy that is intractable with the available treatment options [6].

Our initial report [1] indicated that ONC201 does not significantly induce apoptosis in normal human fibroblasts at doses that trigger pronounced cell death in human cancer cells. Furthermore, several efficacy studies utilized doses of up to 100 mg/kg in mice without incidence of ONC201-related mortality, gross or histological morphological changes, or body weight loss. Immunohistochemical analyses of normal tissues in ONC201-treated mice revealed that TRAIL, which is induced by ONC201 as part of its mechanism, is upregulated at the protein level in the brain, kidney, and spleen of normal mice without apparent toxicity as determined by histology and TUNEL staining. These observations suggest that ONC201 is present at pharmacologically active levels in these normal tissues without causing apparent toxicity at the administered doses.

Since our initial report on ONC201, a series of studies have been undertaken to validate the preclinical profile of ONC201 and facilitate the clinical translation of this unique drug candidate in advanced cancers. Here, we report preclinical studies that confirm the ability of ONC201 to distinguish tumor from normal cells in its cytotoxicity and validate the conservation of this safety feature in other species in GLP toxicology studies. Our findings highlight a wide therapeutic index for ONC201 that may be determined by differences in its effects on cell death receptor signaling between tumor and normal cells.

## Materials and Methods

### Cell culture and reagents

Cell lines were obtained from ATCC and cultured in ATCC-recommended media in a humidified incubator at 5% CO_2_ and 37°C. ONC201 dihydrochloride was obtained from Oncoceutics as previously described [7]. The free-base preparation of the compound can be obtained from the NCI DTP.

### Cell cycle analysis

For cell cycle analysis, cells were trypsinized at the indicated time points and fixed in 80% ethanol at 4°C for a minimum of 30 minutes. Fixed cells were then stained with propidium iodide in the presence of RNase and analyzed on an Epics Elite flow cytometer (Beckman Coulter). Cell viability was assessed using Cell TiterGlo (Promega) in 96-well plates using the manufacturer’s protocol.

### Flow cytometry analysis

For surface TRAIL experiments, adherent cells were harvested by brief trypsinization, fixed in 4% paraformaldehyde in PBS for 20 min, incubated with an anti-TRAIL antibody (Abcam, ab2435) at 1:250 overnight, washed and incubated with anti-rabbit Alexafluor 488 (Invitrogen) for 30 min, and analyzed. Cells were gated on forward and side scatter to eliminate debris and dead cells from the analysis. Surface TRAIL data is expressed as median fluorescence intensity relative to that of control samples unless indicated as otherwise. Surface DR5 was analyzed similarly using an antibody from Imgenex and a similar protocol.

### Animal studies

GLP toxicology studies were performed at Calvert Laboratories in accordance with the U.S. FDA GLP regulations in effect at the time of study conduct (Good Laboratory Practice Regulations, Title 21 Code of Federal Regulations Part 58. All procedures were conducted under a protocol that was approved by Calvert IACUC. Sprague Dawley rats (approximately 10 weeks of age) were obtained from Harlan Sprague Dawley. Beagle dogs (approximately 5–6 months of age) were obtained from Marshall BioResources (North Rose, NY). ONC201 dihydrochloride was formulated in phosphate-buffered saline. Dosing preparations were stored at room temperature following preparation and utilized within 4 hours of completion of preparation.

Animals were dosed once on Day 1 via oral gavage. Mortality/morbidity observations were recorded twice daily and at least once on the day of scheduled euthanasia. Clinical observations were evaluated prior to dose administration and approximately one hour post-dose on dosing days and once daily on non-dosing days. Functional Observational Battery was observed prior to treatment initiation and on Day 1 between 1 and 2 hours post-dose. Body weights were recorded prior to dosing on Day 1 and on Days 2, 7, 14 and 18. Food consumption was recorded daily. Electrocardiograms were obtained from all animals prior to treatment initiation and on Day 1 between 2 and 4 hours post-dose. Blood pressure was obtained from all animals prior to treatment initiation and on Day 1 between 2 and 4 hours post-dose. Blood for evaluation of hematology, coagulation and clinical chemistry and urine for urinalysis was collected prior to dosing and on Days 3 or 19. Blood for toxicokinetic evaluation was collected from all animals at selected time points on Day 1. All animals were sacrificed on Day 3 or Day 19. Selected tissues were harvested at necropsy, selected organs weighed, and selected tissues evaluated microscopically.

## Results

### ONC201 does not induce cell death in normal cells at efficacious doses

**Fig 1:**
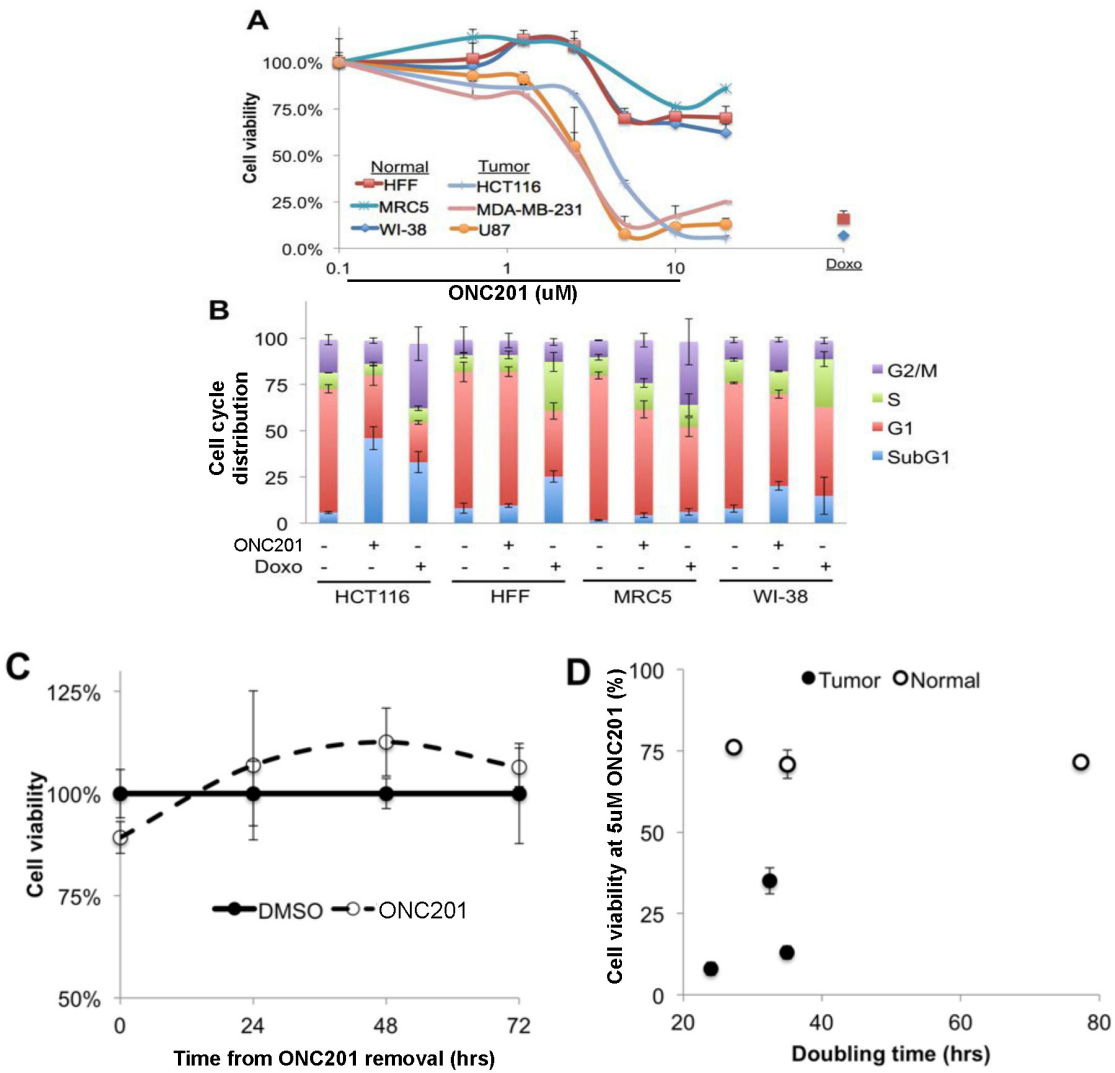
ONC201 effects on proliferation and cell death in tumor and normal cells. (A) Cell viability assays following ONC201 treatment at indicated concentrations for 72 hours (n = 3). Doxorubicin (Doxo) was used at 1 μg/mL as a positive control in normal fibroblasts. (B) Cell cycle analysis of tumor and normal cells following treatment with ONC201 (5 μM) or doxorubicin (Doxo, 1μg/mL) for 72 hours (n = 3). (C) Cell viability assay in MRC5 cells following a 72 hour treatment with ONC201 (5 μM) or DMSO and the indicated recovery period in complete drug-free media after this treatment (n = 3). Time points are given as time following removal on ONC201 after 72 hour treatment. (D) Cell viability of tumor and normal cells following treatment with ONC201 (5 μM, n = 3) for 72 hours.

We assayed the effects of ONC201 on normal human fibroblast proliferation in cell culture following a 72-hour incubation. Comparing dose-response relationships between tumor and normal cell lines revealed a similar inflection point in cell viability assays at ~5 μM that subsequently saturated in all tested cell lines. Interestingly, the effects of ONC201 saturated at much higher magnitudes in tumor cells than normal cells (Fig 1A), i.e. there was little or no toxicity in the normal cells while the tumor cells appeared to completely lose viability at the higher doses. This observation is in sharp contrast to the positive control, doxorubicin, which resulted in massive cell death of both tumor and normal cells. The disparate responses to ONC201 between tumor and normal cells indicate a wide therapeutic index for ONC201 in vitro.

To ascertain if the modest reduction in cell viability observed in normal cells was associated with a reduction in proliferation or an increase in cell death, we investigated the cell cycle profiles of normal cells. We found that ONC201 treatment did not cause any appreciable levels of cell death in normal cells, which are increased in HCT116 tumor cells under the same conditions (Fig 1B). Again by contrast to ONC201, doxorubicin treatment generally resulted in cell death or S-phase arrest in normal cells.

With the observation that ONC201 slightly reduces proliferation in normal cells, we next determined whether this was a reversible phenomenon. We found that removing ONC201 at the end of a 72-hour incubation period results in a time-dependent complete recovery that is evident at 24 hours later (Fig 1C). We investigated the possibility that the difference between tumor and normal cells may simply be a function of proliferation rate. Examining this relationship revealed no obvious correlation between the ability of ONC201 to reduce cell viability and the doubling rate of the cell line (Fig 1D), which are historically correlated with chemotherapy use.

### ONC201 induces TRAIL and DR5 in tumor but not normal cells

**Fig 2:**
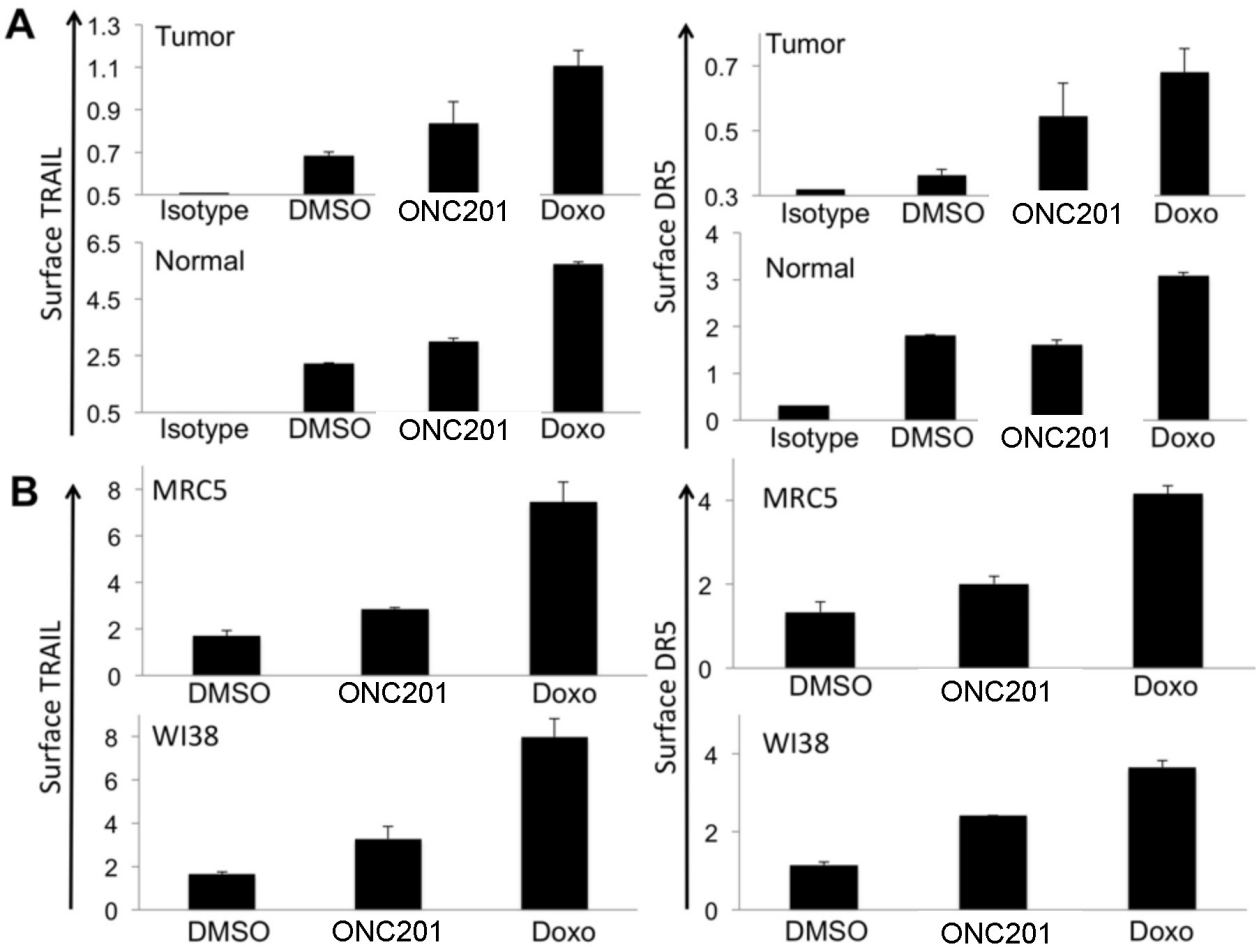
Effect of ONC201 on surface TRAIL and DR5 In tumor and normal cells. (A) Surface TRAIL and DR5 expression in HCT116 (tumor) and HFF (normal) cells following treatment with ONC201 (5 μM, 72 hr, n = 3). IgG was included as an isotype negative control. Doxorubicin (Doxo) is shown as a positive control (1 μg/mL). (B) Surface TRAIL and DR5 in normal fibroblasts following treatment with ONC201 (5 μM) or doxorubicin (1 μg/mL, 72 hr, n = 3). Values (y-axis) is expressed as mean fluorescence intensity.

To provide a molecular explanation for the preferential killing of tumor but not normal cells by ONC201, we investigated the effect of ONC201 on normal cell production of TRAIL and DR5 that are upregulated in response to ONC201 in tumor cells [1]. We found that ONC201 is capable of upregulating surface TRAIL in tumor and normal cells, though to a lesser magnitude in normal cells (Fig 2A). Interestingly, DR5 induction was minimally altered in normal fibroblasts as opposed to tumor cells that boosted production of the death receptor in response to ONC201 (Fig 2A).

Unlike ONC201, doxorubicin boosted surface TRAIL as well as DR5 proteins at similar magnitudes in tumor and normal cells, presumably through p53 that regulates both of these genes [8, 9], thereby demonstrating no appreciable differential activity that is indicative of its narrow therapeutic index. Investigating the effects of ONC201 and doxorubicin on surface TRAIL and DR5 in other normal fibroblasts confirmed these observations (Fig 2B). These studies may explain why ONC201 generally does not induce cell death in normal fibroblasts, which may be linked to the selective apoptotic activity of TRAIL as well as the general absence of DR5 induction in normal cells.

### GLP toxicology studies in ONC201-treated Sprague Dawley rats

To confirm the safety profile of ONC201 and enable clinical testing, GLP toxicology studies were conducted in male and female Sprague Dawley rats with single oral doses of ONC201. Rats received 0, 12.5, 125, or 225 mg/kg ONC201 by oral gavage, which represents a human equivalent of 0, 125 mg, 1.25 g, and 2.25 g, respectively (Table 1). The low-dose rat cohort of 12.5 mg/kg corresponds to ~25 mg/kg in mice that has demonstrated efficacy in preclinical models [1]. There where no mortalities or dose-limiting toxicities in these studies. There was no laboratory or clinical signs of toxicity noted at single doses up to 125 mg/kg of ONC201 in rats. At a dose of 225 mg/kg there were instances of decreased activity and abnormal gait and stance in rats that resolved within a day. A decrease in body weight gain and food consumption was noted on Day 7 for the 225 mg/kg in male but not female rats.

**Table 1:** Cohorts used in GLP toxicology studies with ONC201.

	Group	ONC201	ONC201 Human Equivalent Dose	Number of Animals	
		(mg/kg)	(mg)	Male	Female
Sprague Dawley Rats	Control	0	0	20	20
	Low Dose	12.5	125	10	10
	Mid Dose	125*	1250*	20	20
	High Dose	225	2250	20	20
Beagle Dogs	Control	0	0	6	6
	Low Dose	4.2	125	3	3
	Mid Dose	42*	1250*	6	6
	High Dose	120	3570	6	6

Human equivalent doses are estimated by standard allometric scaling and assumes a 60kg human body weight.

* Indicates the dose determined to be the NOAEL is shaded gray for each species.

There were no significant changes in blood or urine tests. Hematology parameters analyzed included red blood cell count and morphology, white blood cell count, mean corpuscular hemoglobin, mean corpuscular hemoglobin concentration, mean corpuscular volume, hematocrit, hemoglobin, and reticulocyte count. Total and differential white blood cell counts, including neutrophils, basophils, eosinophils, monocytes, lymphocytes and large unstained cells. Coagulation parameters analyzed included activated partial thromboplastin time and prothrombin time. Serum clinical chemistry included alanine aminotransferase, albumin, albumin/Globulin ratio, alkaline phosphatase, aspartate aminotransferase, calcium, chloride, cholesterol, creatinine, globulin, glucose, phosphorus, potassium, sodium, total bilirubin, total protein, trigerclyrerides, and urea nitrogen. Urinanalysis parameters analyzed included specific gravity, pH, protein, glucose, ketone, urobilinogen, nitrite, appearance/color, bilirubin, blood, leukocytes, and microscopic examination of formed elements.

There were also no significant gross organ findings attributable to treatment. ONC201-related minimal-to-mild submucosal edema and/or mixed cell inflammation was observed in the non-glandular stomach of rats treated with 225 mg/kg at two days following treatment but was fully recoverable.

The oral administration of ONC201 at 12.5 and 125 mg/kg did not induce any biologically relevant effects on respiratory rate, tidal volume or minute volume in conscious male rats. A marginal to moderate transient decrease in respiratory rate and minute volume was observed following the oral administration of ONC201 at 225 mg/kg that was reversible within 1 hour of administration and completely recovered within 2 hours of administration.

Based on the results of this study, the NOAEL following oral administration of ONC201 at single doses of 12.5, 125 or 225 mg/kg to Sprague-Dawley rats is considered to be 125 mg/kg, which corresponds to a dose of ~1.25g in humans assuming standard allometric scaling.

### GLP toxicology studies in ONC201-treated beagle dogs

As a second species, GLP toxicology studies were conducted in male and female beagle dogs with single oral doses of ONC201 in parallel to the rat studies. Beagle dogs received a single dose of 0, 4.2, 42, or 120 mg/kg by oral gavage, which represents a human equivalent of 0, 125 mg, 1.25 g, and 3.57 g, respectively. The low-dose dog cohort of 4.2 mg/kg corresponds to ~25 mg/kg in mice that has demonstrated efficacy in preclinical models [1]. There where no mortalities or dose-limiting toxicities in these studies. No clinical signs of toxicity were noted following a single dose of 4.2 mg/kg ONC201. Clinical findings included decreased activity, emesis, vomitus, salivation, and/or soft, loose or mucous feces, and changes in fecal excretion at higher doses that occurred only on the day of drug administration. There were no definitive ONC201-related effects on group mean body weight or body weight gain. The 120 mg/kg dose in female dogs had statistically significantly decreased group mean food consumption as compared to the vehicle control group on Days 14, 15 and 18.

No clear ONC201-related histological changes were noted at the Day 3 or Day 19 necropsy. There were no ONC201 effects on EKG rhythm or morphology in dogs, mean heart rate, arterial blood pressure, hematology, coagulation or clinical chemistry parameters or erythrocyte morphology, urinalysis, necropsy, or organ weights. Based on the results of this study, the NOAEL following oral administration of ONC201 at single doses of 4.2, 42 or 120 mg/kg to Beagle dogs is considered to be at least 42 mg/kg, which corresponds to a dose of ~1.25 g in humans assuming standard allometric scaling.

## Discussion

ONC201 is a first-in-class small molecule targeted anticancer compound under development by Oncoceutics to treat advanced cancer. ONC201 is a stable, orally active, blood brain barrier-penetrable compound that has demonstrated p53-independent anti-cancer activity as a monoagent and a combination in several preclinical models that include glioblastoma, breast cancer, colon cancer, lung cancer, lymphoma, and other tumors [1, 10, 11]. The extensive preclinical studies presented here confirm the ability of ONC201 to exert its anti-tumor effect without harming normal cells, which is an important and unique feature of ONC201 as a potential new cancer therapy.

ONC201 possesses intriguing differences when comparing dose-response curves from tumor versus normal cells that is distinct from other therapies. The overall reduction in cell viability is ~80% in tumor cell lines as opposed to ~20% in normal cell lines reflects a wide therapeutic index. Interestingly, both the tumor and normal cell dose response curves have the same inflection point (~5 μM) and are saturable. However the response in cancer cells is cytotoxic and irreversible whereas that of normal cells is mildly anti-proliferative and reversible. This unique dose-response relationship suggests that ONC201 may engage a molecular target(s) that is/are particularly germane to cancer cells. The most proximal molecular binding target of ONC201 remains an active area of investigation.

ONC201 causes a transient inhibition of proliferation in normal cells that is reversible upon drug removal as opposed to chemotherapy, which induces cell death. While ONC201 induces TRAIL in both normal and tumor cells, DR5 induction is only observed in tumor cells. This key observation suggests that normal cells may not undergo a cytotoxic response to ONC201 as they are naturally TRAIL-resistant [12] and do not increase DR5 expression as tumor cells do. The chemotherapy doxorubicin also induces TRAIL & DR5, presumably through p53 [3, 8, 9], but this occurs in both tumor and normal cells and may explain the cell death observed in normal cells following doxorubicin exposure. The mechanism of ONC201-induced DR5 appears to be transcriptional based on prior studies in human colon cancer cells, however the molecular mechanism of this induction is an area of current study. Lack of DR5 induction may be linked to an absence of CHOP upregulation [13] or Foxo3a activation that both have been implicated in the mechanism of ONC201 [1, 14] and are both transcriptional regulators of the human DR5 gene [13, 15]. Furthermore, the lack of DR5 induction by normal cells may be particularly important for protection from cytotoxicity as a recent report has found that DR5 can induce apoptosis in a TRAIL-independent in some contexts [16]. One limitation of these in vitro studies is the disparate tropism of the cell lines that were evaluated, however these observations are consistent across different tissue types represented by the cell lines in this study with malignant or normal cells.

The safety profile of ONC201 in GLP safety studies is consistent with the preferential cytotoxicity of ONC201 in tumor over normal cells in vitro. Adverse events associated with exaggerated doses of ONC201 are generally mild and reversible. The only findings that were observed in both rats and dogs in GLP toxicology studies occurred at high doses and included decreased activity, decreased food consumption (weight loss only seen in rats), and salivation. The NOAEL doses for both evaluated species were at least 10-fold above the therapeutic dose of ONC201 in prior preclinical studies. Thus the in vitro and in vivo profiles of ONC201 indicate a wide therapeutic index that is uncommon for most cancer therapeutics and implies that ONC201 is well suited to serve as a chronic therapy, and as a well-tolerated drug. This is further supported by a lack of apparent genotoxicity in ONC201-treated cells [10], ONC201 also displays a lack of resistance that is likely a consequence of sharp dose-response curves in tumor cells that confounds the evolution of drug resistance and efficacy in the face of common mechanisms of resistance such as PgP overexpression, cancer stem cells, and genetic aberrations [14, 17].

Based on results of the GLP toxicology studies, the starting dose for the first-in-man clinical trial was calculated to be 125 mg, which is 1/10^th^ the lowest NOAEL in accordance with regulatory guidance documents. The clinical introduction of ONC201 at a dose of 125 mg is promising, given that ONC201 has demonstrated anti-tumor activity in mouse models at this dose and that a >10-fold safety window exists above this dose. Should the safety features and the convenient oral schedule of ONC201 translate to the clinic, a range of clinical settings in oncology may be available to ONC201 that cannot be addressed by current therapies based on their unbearable toxicities, genotoxicity-induced secondary malignancies, and/or rapid induction of resistance.
