# Correction: First-In-Class Small Molecule ONC201 Induces DR5 and Cell Death in Tumor but Not Normal Cells to Provide a Wide Therapeutic Index as an Anti-Cancer Agent

**DOI:** 10.1371/journal.pone.0149365

**Published:** 2016-02-10

**Authors:** Joshua E. Allen, Roslyn N. Crowder, Wafik S. El-Deiry

The second author’s name appears incorrectly. The correct name is Roslyn N. Crowder. The correct citation is: Allen JE, Crowder RN, El-Deiry WS (2015) First-In-Class Small Molecule ONC201 Induces DR5 and Cell Death in Tumor but Not Normal Cells to Provide a Wide Therapeutic Index as an Anti-Cancer Agent. PLoS ONE 10(11): e0143082. doi:10.1371/journal.pone.0143082
